# 4H12, a Murine Monoclonal Antibody Directed against Myosin Heavy Chain-9 Expressed on Acinar Cell Carcinoma of Pancreas with Potential Therapeutic Application

**DOI:** 10.52547/ibj.25.5.310

**Published:** 2021-08-18

**Authors:** Sima Balouchi-Anaraki, Simin Ahmadvand, Akbar Safaei, Abbas Ghaderi

**Affiliations:** 1 Department of Immunology, School of Medicine, Shiraz University of Medical Sciences, Shiraz, Iran;; 2 Shiraz Institute for Cancer Research, School of Medicine, Shiraz University of Medical Sciences, Shiraz, Iran;; 3 Department of Pathology, School of Medicine, Shiraz University of Medical Sciences, Shiraz, Iran

**Keywords:** Acinar cell carcinoma, Biomarkers, Monoclonal antibody, Pancreas

## Abstract

**Background:**

PACC is a rare type of pancreatic exocrine neoplasm that is frequently diagnosed at late stages with a high rate of metastasis. Identification of new biomarkers for PACC can improve our knowledge of its biology, early detection, or targeted therapy. In this study, hybridoma technology was used to generate mAbs against Faraz-ICR, a pancreatic acinar cell carcinoma cell line.

**Methods:**

Cell ELISA and flow cytometry were used for screening, and the 4H12 hybridoma clone was selected for further analysis. The 4H12 mAb was specific for MYH9 as determined by Immunoprecipitation, Western blot, and mass spectrometry.

**Results:**

This antibody reacted variably with other cancer cells, in comparison to Faraz-ICR cell. Besides, by immunohistochemical staining, the acinar cell tumor, which was the source of Faraz-ICR, showed high MYH9 expression. Among 21 PDAC cases, nine (42.8%) expressed MYH9 with low intensity, while 10 (47.8%) and 2 (9.5%) cases expressed MYH9 with moderate to strong intensities, respectively. The 4H12 mAb inhibited the proliferation of Faraz-ICR cells in a dose-dependent manner from 0.75 to 12.5 μg/ml concentrations (*p < *0.0001 and *p *< 0.002). IC_50 _values were achieved at 12.09 ± 4.19 µg/ml and 7.74 ± 4.28 µg/ml after 24- and 48-h treatment, respectively.

**Conclusion:**

Our data suggest that the 4H12 mAb can serve as a tool for investigating the role of MYH9 pancreatic cancer biology and prognosis.

## INTRODUCTION

Pancreatic cancer, one of the most aggressive forms of tumor, is the 11^th^ most common cancer in the world and the 7^th^ leading cause of cancer-related deaths^[^^1^^]^. Among pancreatic malignancies, PACC is a rare form of exocrine tumor, which accounts for about 1-2% of pancreatic malignancies in adults and 15% of pediatric cases^[^^2^^-^^4^^]^. The long-term survival of patients with PACC is higher than those with PDAC^[^^3^^]^. However, PACC is still highly malignant with poor prognosis^[^^5^^]^. Depending on the stage of disease, the median survival time of patients with PACC is about 18-36 months^[^^2^^,^^6^^]^. Like PDAC, surgical resection is the main treatment for PACC patients with localized tumor^[^^7^^,^^8^^]^. There is no specific therapeutic approach for patients with progressive disease when surgery is impossible^[^^3^^,^^9^^]^. Therefore, the discovery of new biomarkers is necessary for the early detection and treatment of pancreatic cancer.

Biomarkers are used as molecular indicators for cancer diagnosis, therapeutic interventions, and the prediction of outcome^[^^10^^,^^11^^]^. Genomics^[^^12^^]^, proteomics^[^^13^^,^^14^^]^, metabolomics^[^^15^^]^, and mAb-based technologies^[^^16^^]^ are employed to detect and confirm new biomarkers^[^^12^^,^^15^^,^^16^^]^. Antibody-based methods have potential to identify new targets that cannot be detected by genomics or proteomics alone, such as conformational epitopes or those undergo post-translational modifications^[^^17^^]^. On the other hand, mAb-based methods, in which the whole cell is applied as an immunogen, are excellent approaches to distinguish tumor-specific antigens in their native forms as expressed in the tumor cells^[^^17^^,^^18^^]^. Moreover, the aforesaid methods can provide valuable information about antigen localization and molecular interactions that may act as key factors in diagnosis, vaccine development, and targeted therapies^[^^19^^,^^20^^]^. 

Among the biomarkers introduced for pancreatic cancer, CA-19-9 is the only case approved by the US Food and Drug Administration^[^^21^^]^. Due to the low sensitivity and specificity, this biomarker is not suitable for screening and differential diagnosis. However, it can be used only for monitoring response to therapy and the prognostic value of PDAC^[^^22^^,^^23^^]^. It has also been shown that CA-19-9 is normal in almost all of the PACCs^[^^24^^]^. Our better understanding of the molecular biology of pancreatic tumors and the identification of new tumor biomarkers can contribute to the development of mAbs, which may lead to the early diagnosis and specific treatment process. 

In this study, we aimed to produce mAb by hybridoma technique against Faraz-ICR cell, which was isolated from a *PACC specimen*^[^^25^^]^*.* After screening the produced hybridoma library, we selected 4H12 clone, and 4H12 mAb was characterized by isotype determination and its reactivity to several cancer cell lines and tumor specimens. Furthermore, its target antigen was determined by mass spectrometry. 

## MATERIALS AND METHODS

**Cell culture**

A panel of human cancer cell lines (MIA PaCa-2, PaTu 8902, MDA-MB-231, MCF-7, SW 1116, and SKOV3) and Sp2/0 myeloma cell were purchased from the National Cell Bank of Iran at the Pasteur Institute of Iran, Tehran. Faraz-ICR cell line was established from a PACC specimen in Shiraz Institute for Cancer Research, Shiraz, Iran^[^^25^^]^. ASCs were isolated from breast fat tissues of healthy individuals underwent mammoplasty^[^^26^^]^. Briefly, adipose tissues were sliced into small pieces and digested by 0.2% collagenase type I (Gibco, USA). Using ficoll (Biosera, USA), stromal cells were isolated by density gradient centrifugation at 400 ×g at 4 °C for 10 minutes. Subsequent to the isolation, the stromal cells were cultured in DMEM (Gibco). After 24 hours, the culture medium was discarded, along with non-adherent cells, and then adherent cells were expanded in a fresh medium. Faraz-ICR, MIA PaCa-2, and PaTu 8902 cell lines and also ASCs were cultured in DMEM. Other cancer cells were cultured in RPMI 1640 (Gibco). The culture medium was supplemented with 10% heat-inactivated fetal bovine serum (Gibco) and 100 U/ml of penicillin, and 100 mg/ml of streptomycin (Sigma-Aldrich, Germany), and the cells were maintained in a CO_2_ incubator at 37 °C until the cells reached 80% confluence. 

**Immunization of mice**

Six-week-old female BALB/c mice, obtained from the Pasture Institute of Iran, were immunized with four repeated intraperitoneally injections of 7 × 10^6 ^Faraz-ICR cells suspended in 500 µl of PBS, administrated at a two-week interval. Mice were bled from the tail vein before the first immunization and one week after each boost, and serum was screened by ELISA. The mouse with the highest response was selected, and the final boost was administrated three days before scarification.

**ELISA**

At first, a 96-well microtiter plate (Nunc Maxisorp, Denmark) was coated with 100 µl of 20 µg/ml of Faraz-ICR cell lysate diluted in carbonate-bicarbonate coating buffer (pH 9.6) at 4 °C overnight. The plate was blocked with 1% bovine serum albumin (Biosera) at 37 °C for two hours. Subsequently, the plate was further incubated with twofold serial dilutions (from 1:100 to 1:4000) of sera at 37 °C for two hours. Pre-immunized sera were used as negative controls. After washing with PBS-Tween (PBS containing 0.05% Tween-20), HRP-conjugated goat anti-mouse immunoglobulin antibody (BD Biosciences, USA) at 1:2000 dilution was added to each well and incubated for one hour. Finally, after adding 3,3',5,5'-Tetramethylbenzidine substrate (Invitrogen, USA), the plate was incubated in the dark at room temperature for 15 minutes; reaction was stopped by the addition of sulfuric acid (0.2 M). The absorbance was measured with a microplate reader (Anthos 2020, Austria) at 450 nm^[^^18^^]^.

**Hybridoma generation**

The mouse with the highest response was scarified three days after the last boost, and the splenocytes were isolated by gently flushing medium through the spleen. The splenocytes were fused with SP2/0 cells in 5:1 ratio by polyethylene glycol 1500 (Sigma-Aldrich) to generate hybridoma. The resulting hybridomas were cultivated in RPMI supplemented with 20% FBS, 1× non-essential amino acids, 1 mM of sodium pyruvate (both from Gibco, UK), and 1× selective HAT medium (Sigma-Aldrich) in 96-well culture plates. The reactivity of hybridoma cell culture supernatant was screened by cell-based ELISA and flow cytometry after 14 days of fusion.

**Cell-based ELISA**

The cell-based ELISA was performed as described previously^[^^27^^]^ with some modifications. Faraz-ICR cells were seeded at 3 × 10^4 ^cells/well onto 96-well plates (SPL Life Sciences, South Korea) and incubated at 37 °C overnight; then the cells were fixed with pre-chilled methanol for 10 minutes. After blocking with 5% bovine serum albumin (Biosera), the cells were incubated with undiluted hybridoma supernatants and re-incubated with HRP-conjugated goat anti-mouse immunoglobulin antibody (BD Biosciences) at 1:1500 dilution for one hour. At the end, after adding 3,3',5,5'-Tetramethylbenzidine substrate (Invitrogen) in the dark for 15 minutes, reaction was stopped with 0.2 M of sulfuric acid. The absorbance was measured with a microplate reader at 450 nm.

**Flow cytometry**

Flow cytometry was performed to evaluate the surface expression of target antigen on leukocytes, ASCs, MIA PaCa-2, PaTu 8902, MDA-MB-231, MCF-7, SW 1116, and SKOV3 cells. Leukocytes were isolated from peripheral blood using a red blood cell lysis buffer (0.17 M of ammonium chloride). For surface staining, 2 × 10^5^ cells were incubated with 50 µl of undiluted hybridoma supernatants (for screening) or purified 4H12 mAb (10 µg/ml) at 4 °C for 30 minutes. The cells were washed with PBS containing 2% FBS, incubated with FITC-conjugated goat anti-mouse immunoglobulin (Sina-Biotech, Iran) at 4 °C for 30 minutes, washed again and acquired on a FACSCalibur flow cytometer (BD Biosciences). For intracellular staining, 5 × 10^5^ cells were incubated with Paraformaldehyde 1% (Sigma-Aldrich) for 15 minutes at 4 °C fallowed by washing with PBS. Cells were incubated with perm/wash (BD Biosciences) and then with 50 µl of or purified 4H12 mAb (10 µg/ml) at 4°C for 30 minutes. After additional washes with perm/wash, incubation was performed with FITC-conjugated goat anti-mouse immunoglobulin at 4 °C for 30 minutes. In the final step, washed cells were subjected to flow cytometry, and data were analyzed using FlowJo software (version 7.6.2, Ashland, San Diego CA, USA).

**Determining the mAb isotype**

Selected hybridoma was cloned three times by the limiting-dilution method, and its isotype was determined using hybridoma supernatant and Mouse Ig Isotyping ELISA Ready-Set-Go!™ Kit (Invitrogen, Austria) according to the manufacturer's instructions.

**mAb purification**

Purification of 4H12 mAb from ascitic fluid was performed by affinity chromatography on Hi-Trap protein G column (GE Healthcare, Sweden) in an ÄKTAprime Plus Chromatography System (GE Healthcare, UK). The elution process was carried out using an elution buffer (100 mM of glycine, pH 2.7), followed by pH adjustment to 7.0 with 1 M of Tris-HCl (pH 9.0)^[^^28^^]^. After the dialysis against PBS (pH 7.4) at 4 °C overnight, we determined the concentration of the 4H12 mAb from sample absorbance at 280 nm using the *NanoDrop *2000c Spectrophotometer (Thermo Scientific, USA).

**Immunoprecipitation and antigen identification**

To identify the target antigen, we performed immunoprecipitation using Pierce Crosslink Immunoprecipitation Kit (Thermo Scientific) according to the manufacturer's instructions. In brief, mAb was incubated with protein A/G agarose, and then the pre-cleared Faraz-ICR cell lysate was added to the resin and incubated at 4 °C overnight. After removing the unbound proteins by washing, the specific protein was eluted and subjected to SDS-PAGE and Western blotting. The protein band was extracted and evaluated using LC-MS/MS. 

**Western blotting**

Immunoprecipitated protein was evaluated by SDS-PAGE under reducing conditions on a 10% polyacrylamide gel. Briefly, the sample was mixed with a sample buffer containing ditiotheriol (at the final concentration of 100 mM). Thereafter, the sample was heated at 95 °C for 5 minutes and then allowed to cool to room temperature before loading in gel. After electrophoresis, one gel was stained with colloidal Coomassie Blue, and the other one was used to transfer protein to PVDF membrane (GE Healthcare, Germany) for Western blotting. The membrane was incubated in a blocking solution (PBS-Tween containing 5% skim milk) at 4 °C overnight and then probed with 35 µg/ml of 4H12 mAbs at room temperature for 1 h. After washing with PBS-Tween, the membrane was re-incubated with goat HRP-conjugated anti-mouse antibody (1:4000 in PBS-Tween) for one hour^[29]^. For visualization, Western ECL substrate (Bio-Rad, USA) was used, and protein band was detected with the enhanced chemiluminescence system (Bio-Rad). 

**Immunohistochemistry**

Initially, one PACC and 21 PDACs tissue samples (Supplementary Table 1) were collected. Subsequently, the slides of tumor sections from formalin-fixed paraffin embedded tissues were deparaffinized by fresh xylene, followed by rehydration with decreasing graded ethanol. Antigen retrieval step was performed in Tris-EDTA (pH 9) in a pressure cooker for about 10 minutes. After endogenous peroxidase was washed with PBS, its activity was quenched by 10% H_2_O_2_. Goat serum (10%) was applied to block non-specific hydrophobic protein-protein interactions. The 4H12 mAb was added at 10 µg/ml concentration and incubated in a humid chamber at room temperature for 45 minutes. Thereafter, Master Polymer Plus Detection System Peroxidase (Incl.DAB Chromogen; Master Diagnostica, Spain) was used for color development. After counterstaining with hematoxylin, sections were dehydrated in increasing ethanol concentrations, washed in xylene and mounted using permanent mounting medium, respectively (Merck, Germany)^[^^30^^]^.

**Supplementary Table 1:** Patient characteristics, TNM classification, stage and grade of pancreatic cancer and normal samples

**Patients no.**	**Age**	**Sex**	**Diagnosis**	**TNM**	**Stage**	**Grade**
1	58	F	ACC	T1N0MX	IA	I
2	48	M	DA	T2N2M1	IV	I
3	40	M	DA	T1N1M0	IA	I
4	52	M	DA	T1N0M0	IA	I
5	61	F	DA	T1N1M0	IA	II
6	56	F	DA	T3N1M0	IIB	I
7	44	M	DA	T2N1MX	IIB	I
8	84	M	DA	T2N0M0	IA	I
9	52	F	DA	T3N1MX	IIB	I
10	61	F	DA	T3N2MX	IIB	I
11	67	M	DA	T3NIM0	IIB	II
12	67	M	DA	T2N1M0	IIB	II
13	51	F	DA	T2N0M0	IB	I
14	56	F	DA	T2N0M0	IB	I
15	43	F	DA	T1N0M0	IA	II
16	63	M	DA	T2N1M0	IIB	II
17	77	M	DA	T1N1M0	IIB	I
18	52	M	DA	T2N1M0	IIB	II
19	48	M	DA	T2N1M0	IIB	II
20	61	M	DA	T2N0M0	IB	II
21	73	F	DA	T2N1M0	IIB	II
22	64	M	DA	T3N1M0	IIB	I
23	58	F	NDT	-	-	-
24	52	M	NDT	-	-	-
25	61	F	NDT	-	-	-
26	56	F	NDT	-	-	-
27	44	M	NDT	-	-	-
28	84	M	NDT	-	-	-
29	67	M	NDT	-	-	-
30	51	F	NDT	-	-	-
31	56	F	NDT	-	-	-
32	43	F	NDT	-	-	-
33	63	M	NDT	-	-	-
34	77	M	NDT	-	-	-
35	48	M	NDT	-	-	-
36	61	M	NDT	-	-	-
37	73	F	NDT	-	-	-
38	40	M	NDT	-	-	-

F, female; M, male; ACC, acinar cell carcinoma; DA, ductal adenocarcinoma; NDT, normal adjacent tissue

**Cell proliferation assay**

Faraz-ICR and MCF-7 cells were seeded onto a 96-well plate (1.5 × 10^4 ^and 8 × 10^3 ^cells/well, respectively) and incubated at 37°C overnight. Cells were treated in triplicate with different concentrations of 4H12 mAb (0, 0.75, 3.12, 6.25, 12.5, 25, 50 µg/ml) and culture media (as negative control) at 37 °C for 24 and 48 hours. Then 100 μL per well of MTT solution [3-(4, 5-dimethylthiazol-2-yl), 5-diphenyltetrazolium bromide] (Sigma-Aldrich) at the concentration of 0.5 mg/ml was added and re-incubated at 37 °C for 4 hours. After removing supernatants, 150 µL of DMSO was added to each well for dissolving formazan crystals (Merck) and incubated in dark for 30 minutes. Colorimetric evaluation was performed at 490 nm^[31]^. The experiment was repeated three times, and the data represented as mean ± SD. The percentage of proliferation inhibition was calculated as follows: 

100 - ([absorbance value of the test-absorbance value of the blank])/[absorbance value of the control-absorbance value of the blank]) × 100

**Statistical analysis**

Statistical analyses were carried out using GraphPad Prism Software (version 6.01). The unpaired two-tailed Student's t-test was used to compare two groups. The analysis of variance (ANOVA) and the post-hoc Bonferroni tests were applied for the multiple comparisons of the groups. Data were presented as mean ± SD, and *p* < 0.05 was considered statistically significant.

**Ethical statement**

Mice were treated according to Animal Welfare Guidelines and Policies of Ethical Committee of Shiraz University of Medical Sciences, Shiraz, Iran. The above-mentioned sampling protocols and mouse experiments were approved by the Research Ethics Committee of Shiraz University of Medical Sciences, Shiraz, Iran. (Ethical code: IR.SUMS.REC.1396.5517). 

## RESULTS

**Production and characterization of mAbs against Faraz-ICR cell**

**Supplementary Fig. 1:**
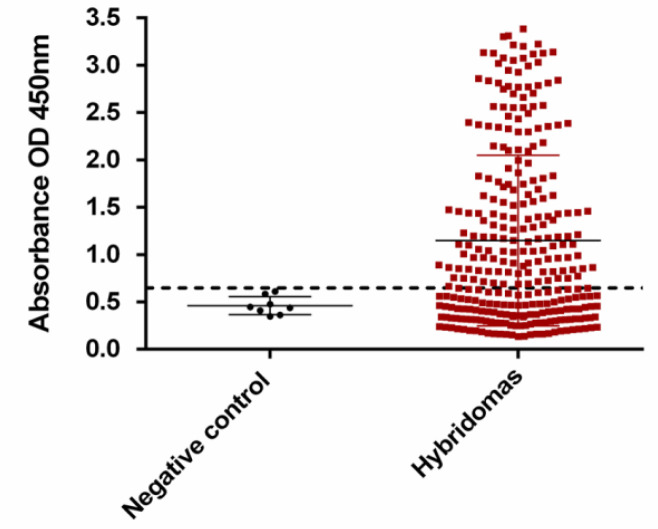
Hybridoma supernatants screening by cell-based ELISA. *Cell-based ELISA was performed using* Faraz-ICR cells and 100 µl of hybridoma supernatant as the source of the primary antibody and HRP-conjugated anti-mouse immunoglobulin secondary antibody. Horizontal dashed line represents negative cut-off (OD = 0.65). Among 376 hybridoma supernatants, 172 showed reactivity with Faraz-ICR cells. Sp2/0 cells supernatant was used as the negative control. Experiment was carried out in triplicates, and data are shown as mean ± SD

**Fig. 1:**
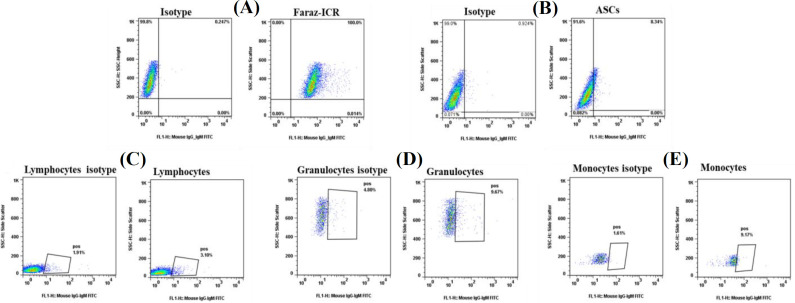
Screening of 4H12 hybridoma supernatants by flow cytometry. Cells were stained with 100 µl of hybridoma supernatant and FITC-conjugated goat anti-mouse immunoglobulin antibody. Cells incubated only with secondary antibody served as the isotype control. The 4H12 hybridoma supernatant showed ~100% reactivity with (A) Faraz-ICR cells and extremely low levels of reactivity with (B) ASCs, (C) lymphocytes, (D) granulocytes, and (E) monocytes

According to the mouse serum titration, the mouse with the highest immune responses at 1:1000 serum dilutions was selected for fusion (data not shown). Numerous hybridomas were produced by the fusion of splenocytes from immunized mice with SP2/0 cells. The hybridoma supernatants were first screened by cell-based ELISA against Faraz-ICR cells. The cut-off value was determined as mean +2SD of negative controls OD, and hybridomas with OD > 0.65 were considered as positive (Supplementary Fig. 1). Positive hybridoma clones (n = 172) were selected for further screening against human leukocyte and mesenchymal stem cells by flow cytometry, respectively (data not shown). According to the flow cytometry results, 4H12 antibody-producing hybridoma, which displayed high reactivity (about 100%) against Faraz-ICR cells and low reactivity against both ASCs (about 8.34%) and leukocytes (about 3.1% of lymphocytes, 9.68% of granulocytes, and 9.7% of monocytes), was selected for further analysis (Fig. 1). After a three-time subcloning, the 4H12 mAb isotype was found to be IgG_2_a κ. 

**Reactivity of purified 4H12 mAb with Faraz-ICR and other cancer cell** **s**

**Fig. 2:**
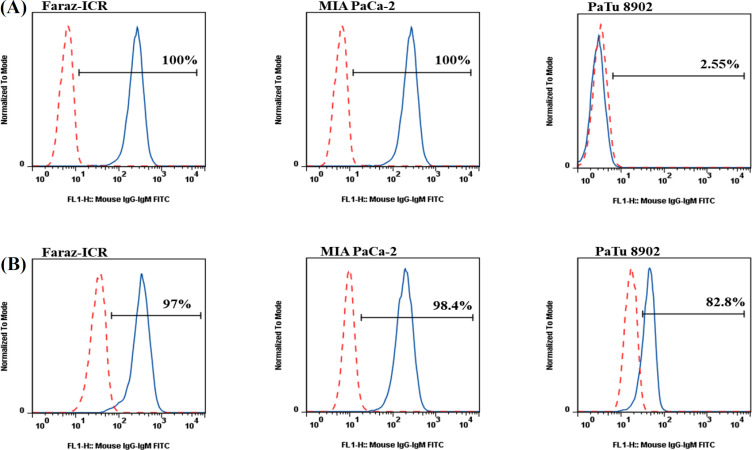
*Flow cytometry evaluation of the reactivity *of 4H12 mAb with human pancreatic cancer cell lines. (A) Extracellular and (B) intracellular staining of Faraz-ICR, MIA-PaCa 2, and PaTu 8902 cells were carried out with 10 µg/ml of 4H12 mAbs and FITC-conjugated goat anti-mouse immunoglobulin secondary antibody (continuous blue lines), and then cells were examined by flow cytometry. Red dashed lines indicate isotype controls

We investigated the reactivity of purified 4H12 mAb with the human pancreatic cancer cell lines, including Faraz-ICR, MIA-PaCa 2, and PaTu 8902 cells by flow cytometry. Surface staining of these cell lines showed that both Faraz-ICR and MIA-PaCa2 had about 100% reactivity with 4H12 mAb compared to isotype control. However, PaTu 8902 cells were found to be negative for 4H12 specific target (Fig. 2A). Intracellular staining of pancreatic cancer cell lines showed the high 

**Fig. 3:**
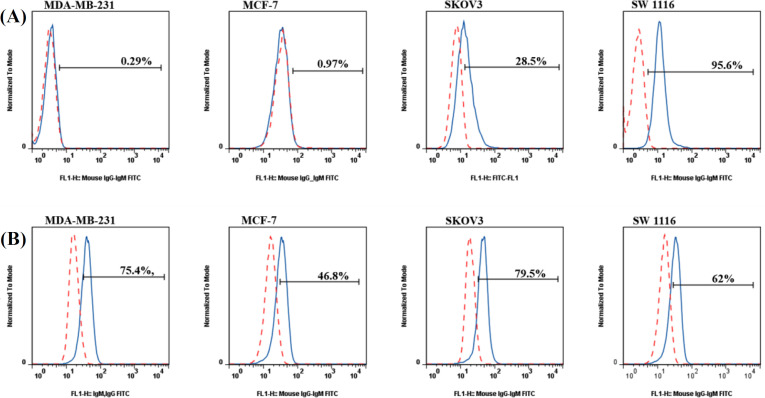
*Flow cytometry evaluation of the reactivity *of 4H12 mAb with different human cancer cell lines. (A) Extracellular and (B) intracellular staining of *MDA-MB-231, *MCF-7, SKOV3, and SW 1116 cancer cells were carried out with 10 µg/ml of 4H12 mAb and FITC-conjugated goat anti-mouse immunoglobulin secondary antibody (continuous blue lines), and then cells were acquired on fa low cytometer. Red dashed lines indicate isotype controls

cell levels of reactivity with 4H12 mAb in Faraz-ICR (98.8%), MIA-PaCa2 (100%), and PaTu 8902 (83.8%; Fig. 2B). Furthermore, we assessed the frequencies of extracellular and intracellular expression of target antigen by other cancer cell lines, including the human breast cancer cells MDA-MB-231 and MCF-7, ovarian cancer cell line SKOV3, and colorectal cancer SW1116 (Fig. 3). Nonetheless, the surface staining of MDA-MB-231 and MCF-7 was found to be negative, and these cell lines showed higher levels of intracellular reactivity with 4h12 mAb (75.4% and 46.8%, respectively). In contrast, SKOV3 (28.5% and 79.5%) and SW1116 (95.8% and 62.5%) indicated the high level of extracellular and intracellular expression of target antigen, respectively.

**Identification of target antigen recognized by 4H12 mAb**

**Fig. 4:**
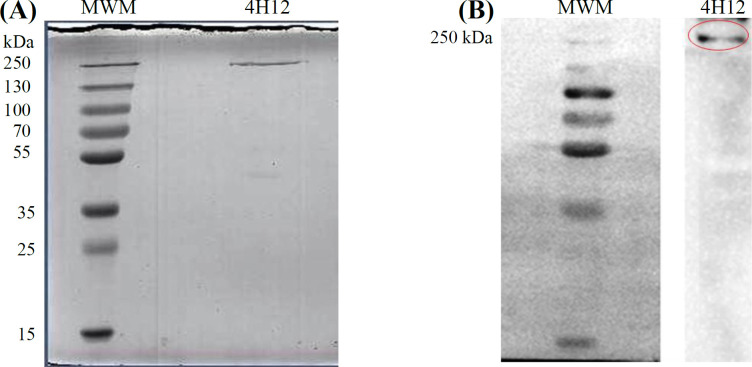
Immunodetection of 4H12 mAb target antigen. (A) Immunoprecipitation was performed with 4H12 mAb. SDS-PAGE shows a protein band of about 250 kDa. (B) Immunoprecipitated antigen was probed with 4H12 mAb. MWM, molecular weights marker

For the identification of target antigen, immuno-precipitation was performed, followed by SDS-PAGE, Western blotting, and LC-MS/MS analysis. The 4H12 mAb immunoprecipitated a band with the molecular weights of about 250 kDa (Fig. 4A). The molecular weight of the target antigen was further evaluated by Western blotting using 4H12 mAb as the primary antibody (Fig. 4B). For the determination of target antigen, the specific band was excised from colloidal Coomassie staining gel and analyzed by LC-MS/MS mass spectrometry. The band was identified as myosin-9, which also coined as MYH9 protein or NMHCIIA (Table 1).

**Immunohistochemical detection of 4H12 mAb target antigen**

**Fig. 5:**
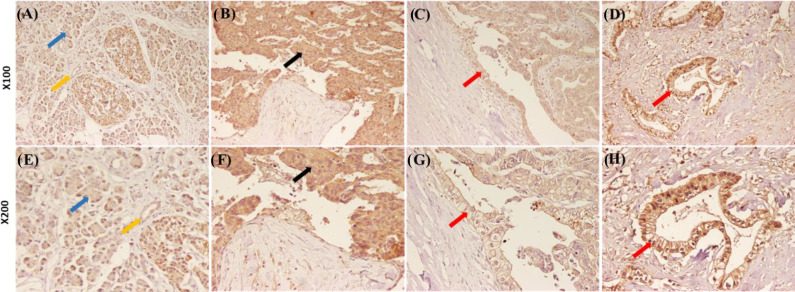
Immunohistochemical staining of pancreatic cancer tissues. (A) and (E) Moderate cytoplasmic MYH9 expression by normal acinar cells (blue arrows) and normal duct (yellow arrows). (B) and (F) The immunohistochemistry staining pattern of MYH9 protein on the acinar cell carcinoma tissue that Faraz-ICR cell originated from. Tumour acinar cells are highly positive for MYH9 expression (black arrows). (C), (D), (G), and (H) show low and high cytoplasmic/membranous MYH9 expression in tumour ducts, respectively (red arrows). The 4H12 mAb has been used at 10 µg/ml concentration. Images have been taken in ×100 and × 200 magnifications

The expression of MYH9 was assessed by 4H12 mAb in resected specimens of one PACC and 21 PDACs (Supplementary Table 1). In our study, only one acinar cell carcinoma tissue was the source of Faraz-ICR cell line, which showed high MYH9 expression (Fig. 5B and 5F) intensity as compared with the normal tissue (Fig. 5A and 5E) and was used as a positive control to set up the immunohistochemistry. All of the 21 ductal adenocarcinoma cases were positive for MYH9 cytoplasmic expression with low (n = 9, 42.8%), moderate (n = 10, 47.6%), and high (n = 2, 9.5%) intensities (Fig. 5C and 5D). However, in ductal adenocarcinoma, six (28.6%) cases showed both membranous and cytoplasmic staining pattern from moderate (n = 5) to high (n = 1), as represented in Figure 5C and 5D. In 15 normal adjacent tissues, ductal cells showed to be weak (n = 10, 66.7%) and moderate (n = 5, 33.3%). None of the normal ductal cells indicated strong expression intensity. It should be mentioned that 95% of the stromal cells were negative for MYH9 expression.

**Effect of 4H12 mAb on cell proliferation**

**Fig. 6:**
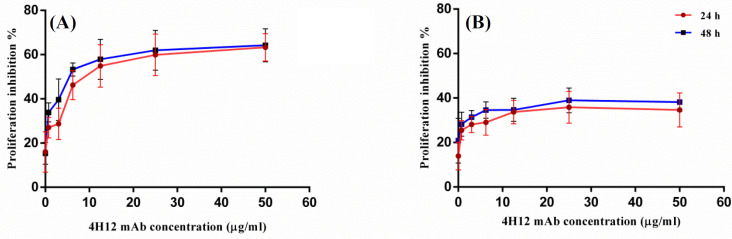
Anti-proliferative effect of 4H12 mAb on (A) Faraz-ICR and (B) MCF-7 cells using MTT assay. Cells were treated with mAbs (0 to 50 µg/ml) for 24 and 48 hours. Experiment was carried out in triplicates and repeated three times. Data are shown as mean ± SD

Effects of 4H12 mAb on the proliferation of Faraz-ICR and MCF-7 cells were studied as a time- and dose-dependent experiment after 24 and 48 hours, at the concentrations of 0.75-50 µg/ml. MCF-7 cell was used because among tested cell lines, it showed the least reactivity with 4H12 mAb. Proliferation of Faraz-ICR cell was significantly inhibited by 4H12 mAb in a dose-dependent manner in 24 and 48 hours with 63.27 ± 6.1% and 64.21 ± 4.3% maximum inhibition, respectively (Fig. 6A). After 24 hours, the maximum inhibition (63.27 ± 6.1% at 50 µg/ml) displayed statistically significant difference from the percentage of inhibition at the concentrations of 12.5 µg/ml (54.9 ± 9.5%; *p* < 0.002), 6.25 µg/ml (46.20 ± 6.59%; *p* < 0.0001), 3.1 µg/ml (28.67 ± 7.12%; *p* < 0.0001), and 0.75 µg/ml (27 ± 4.65%; *p *< 0.0001). In Faraz-ICR cell, the averages of IC_50_ of the mAb were achieved at 12.09 ± 4.19 µg/ml and 7.74 ± 4.28 µg/ml after 24 and 48 hours, respectively, and this difference was not statistically significant (*p* = 0.40). There was no significant proliferation inhibition in the MCF-7 cell (IC_50_ > 50 µg/ml), and only 38% proliferation inhibition was observed, even after 48 hours of treatment with 4H12 mAb (Fig. 6B).

## DISCUSSION

Due to high recurrence rate and incidence of metastases either at or after diagnosis, PACC is considered a cancer with poor prognosis^[^^3^^,^^32^^,^^33^^]^. Moreover, because of low incidence rate, little knowledge is available about PACC, which are mainly derived from sporadic case reports. Therefore, there is an urgent need to find new biomarkers for this type of cancer to improve screening, early diagnosis, and targeted therapy. In this study, using hybridoma technology, a mouse mAb was generated against MYH9 protein expressed by a human acinar cell carcinoma cell line, Faraz-ICR. 

MYH9 encoded-protein, NMHCIIA, normally plays a key role in cell-cell adhesion, proliferation, and migration^[^^34^^]^. It was first discovered in a number of autosomal dominant diseases, in which genetic mutations in MYH9 gene resulted in thrombo-cytopenia^[^^35^^,^^36^^]^. There are controversial data about the role of MYH9 protein in cancer. A few studies on squamous cell carcinoma considered this protein as a tumor suppressor^[^^37^^]^, whereas MYH9 overexpression in various malignancies such as pancreatic cancer^[38]^ esophageal squamous cell carcinoma^[^^39^^]^, lung carcinoma^[^^40^^]^, and acute myeloid leukemia^[^^41^^]^ was associated with aggressiveness, poor prognosis, and reduction in therapeutic response. Watanabe and colleagues^[^^42^^]^ reported the extraction of non-muscular myosin from pancreatic acinar carcinoma cells and stated that the subunits of this molecule undergo phosphorylation by post-translational modification.

**Table 1:** Identification of proteins recognized by 4H12 mAb by LC-MS/MS

mAb	Unused	%Cov (95)	Accession #	Protein name	Peptides (95%)
**4H12**	234.43	53.7	sp|P35579|MYH9_HUMAN	Myosin-9 OS=Homo sapiens GN=MYH9 PE=1 SV=4	295

Unused: the score computed by the software according to the number of good peptides (the higher the score, the higher the confidence); %Cov: coverage percentage; accession: protein identification tag; peptides (95%): number of the identified peptides with a confidence higher than 95%.

In this study, 4H12 mAb showed different reactivity with cancer cell lines as determined by flow cytometry. Interestingly, we observed that the antigen recognized by 4H12 mAb overexpressed on the surface and in cytoplasm of two pancreatic cell lines, including Faraz-ICR and a colon cell line. However, in other cell lines derived from breast and ovarian cancers, the intracellular expression was obviously higher than the surface expression. According to our knowledge, no previous study has demonstrated the expression of MYH9 at the surface of cancer cells using flow cytometry. Nonetheless, it has been indicated that membranous expression of MYH9 facilitates viral infections, by acting as a receptor for sialylated RNA viruses^[^^43^^,^^44^^]^.

In immunohistochemistry staining of the tumor from which Faraz-ICR cell was derived, high cytoplasmic staining of the acinar tumor cells with 4H12 mAb was observed. Our results may indicate an increase in the number of ductal adenocarcinoma specimens that show the moderate and high intensity of MYH9 expression in comparison with normal tissues, which signifies the importance of further evaluation in greater sample size. Moreover, only 28.6% of all PDAC cases demonstrated both membranous and cytoplasmic staining. A study on non-small cell lung cancer indicated MYH9 expression in both cytoplasm and cell membrane of about 38% of cases^[^^45^^]^. Based on MYH9 protein role in cell polarity and protrusion of the lamelliapodia at the leading edge of the cell, it was suggested that membranous staining may be indicative of its role in cancer cell invasion and migration^[^^45^^]^. Another study disclosed that membranous and cytoplasmic expression of MYH9 in esophageal squamous cell carcinoma was definitely associated with lymph node metastasis, serosal invasion, and stage of the disease^[^^39^^]^. Therefore, the surface and intracellular expression of MYH9 in PACC and PDAC should be further evaluated in a larger sample size, and the correlation of either surface or cytoplasmic expression with pancreatic cancer outcome should be determined. 

Our results showed that 4H12 mAb can inhibit the proliferation of Faraz-ICR cells in a dose-dependent manner. Recently, Zhou *et al.*^[^^38^^]^ have investigated the function of non-muscle myosin IIA in pancreatic cancer. They reflected that the knockdown of non-muscle myosin IIA inhibited the proliferation of pancreatic cancer cells, migration and invasion through controlling Wnt/β-catenin by inhibiting nuclear translocation of β-catenin and suppressing its transcriptional activity. Likewise, another study on hepatocellular carcinoma has revealed that an MYH9/GSK3β/β-catenin/c-Jun regulatory circuit improves cancer stemness, migration, invasion, and resistance to sorafenib. MYH9 silencing, significantly improved the survival of mice by enhancing the sensitivity to treatment^[^^46^^]^. It has been reported that MYH9 significantly increases tumorigenesis in colorectal cancer through the activation of MAPK/AKT signaling pathway, which mediates the epithelial mesenchymal transition and correlates with poor prognosis. Furthermore, MYH9 may be a biomarker for diagnosis and a target for the treatment of colorectal cancer^[^^47^^]^.Whether 4H12 mAb has a blocking effect on MYH9 in acinar cell carcinoma cell or can inhibit its growth and metastasis needs further *in vitro* and *in vivo* studies. 

We produced a mAb against MYH9 protein selected from a panel of antibodies against a human PACC cell line. This antibody can be used as a tool to study the role of MYH9 in the biology of pancreatic cancer. By immunohistochemical staining of more tissue samples of acinar and ductal carcinoma of the pancreas, the correlation of MYH9 with cancer outcome and patients’ survival can be determined. 
